# Heterochrony of puberty in the European badger (*Meles meles*) can be explained by growth rate and group-size: Evidence for two endocrinological phenotypes

**DOI:** 10.1371/journal.pone.0203910

**Published:** 2019-03-06

**Authors:** Nadine Adrianna Sugianto, Chris Newman, David Whyte Macdonald, Christina Dagmar Buesching

**Affiliations:** Wildlife Conservation Research Unit, Department of Zoology, University of Oxford, Oxford, United Kingdom; University of Sassari, ITALY

## Abstract

Puberty is a key stage in mammalian ontogeny, involving endocrinological, physiological and behavioural changes, moderated by intrinsic and extrinsic factors. Thus, not all individuals within one population achieve sexual maturity simultaneously. Here, using the European badger (*Meles meles*) as a model, we describe male testosterone and female oestrone profiles (using Enzyme-immunoassays) from first capture (3 months, post-weaning) until 28 months (attaining sexual maturity and final body size), along with metrics of somatic growth, scent gland development and maturation of external reproductive organs as well as intra-specific competition. In both sexes, endocrinological puberty commenced at ca. 11 months. Thereafter, cub hormone levels followed adult seasonal hormone patterns but at lower levels, with the majority of cubs reaching sexual maturity during their second mating season (22–28 months). Interestingly, there was evidence for two endocrinological phenotypes among male cubs (less evident in females), with early developers reaching sexual maturity at 11 months (first mating season) and late developers reaching sexual maturity at 22–26 months (second mating season). Early developers also attained a greater proportion of their ultimate adult size by 11 months, exhibiting faster growth rates than late developers (despite having similar adult size). Male cubs born into larger social groups tended to follow the late developer phenotype. Our results support the hypothesis that a minimum body size is required to reach sexual maturity, which may be achieved at different ages, even within a single population, where early maturity can confer individual fitness advantages and enhance population growth rate.

## Introduction

Puberty involves a variety of endocrinological, physiological, and behavioural changes in mammals [1], resulting in the first occurence of ovulation/oestrus in females and the onset of spermatogenesis in males [2], along with the development of other secondary sexual characteristics [3]. Typically both sexual and somatic maturation are completed [4], but some species continue to grow even after reaching sexual maturity [5]. Although age at puberty depends predominantly on intrinsic genetic factors, its timing can also be moderated by extrinsic factors, such as food availability, seasonal variation, environmental conditions [6–8] the presence of conspecifics [2], and dynamic interactions between these factors [4]. Consequently, not all members of a species [9], or even all individuals within one population [1, 10–13] mature simultaneously or at the same rate, leading to heterochrony [14–15]. In mammals, onset of puberty often depends on attaining a minimum body size (a functional proportion of the final adult body size [10,16]), which conspecifics may attain at different ages (e.g., dairy calves require 56–60% adult body weight attained between 49.8 and 58.2 weeks of age: [10]). Individuals experiencing restricted resources during development due to poorer nutritional condition tend to undergo puberty at a relatively older age [13], leading to a trade-off between somatic growth and puberty/ reproductive activity [17].

Endocrinologically puberty entails the full activation of the hypothalamic-pituitary-gonadal (HPG) axis. This commences the episodic release of gonadotropin releasing hormones (GnRH) by the hyphothalamus, in turn activating the anterior pituitary gland to secrete luteinizing hormone (LH) and follicle stimulating hormone (FSH) that instigate the generation of gamets and release of sex steroids [18]. In males LH stimulates testosterone production from testicular interstitial cells (Leydig cells), and FSH stimulates testes growth and enhances production of an androgen-binding protein by the Sertoli cells within the testicular tubules, necessary for sustaining maturing sperm [3]. In females, FSH stimulates the ovarian follicle(s), causing one/ several ovum/ ova to grow, and triggers follicular oestrogen production. This rise in oestrogen causes the pituitary gland to cease FSH production and instead increase LH production, which in turn causes the ovum/ ova to be released from the ovary, resulting in ovulation [3]. Oestrogen and testosterone levels are therefore low throughout the prepubertal period, but increase immediately prior, during and after puberty, toward adult concentrations [19–20].

Despite numerous studies [6, 8, 21], for many mammals the factors driving sexual development and potential heterochrony in puberty remain incompletely understood, especially for wild-living populations [7, 20–21]. A difficulty is that in most carnivores (for exceptions see [22–24]), and all mustelids [25], seasonal reproductive quiescence (when males cease spermatogenesis and females do not undergo oestrus cycles [26–27]) affects ability to determine the timing of their sexual maturity [24]. Here we use the European badger (*Meles meles*; henceforth “badger”) as a model seasonally breeding carnivore to investigate endocrinological changes and concomitant ontological development during puberty. We examine how onset of puberty can be affected by body size and intra-specific competition resulting in developmental heterochrony.

### Badger reproduction and development

The badgers’ mating season is typically restricted mainly to January-March (but see [28]), although further matings can occur throughout the summer [29]. Local population density determines the number of additional oestrous cycles, ranging from nil to monthly [30–31]. Scent marking activity increases during the mating season [32], reflected by significant elevation in the production of subcaudal (scent) secretion [33] as well as changes to the secretion’s chemical composition [34]. This secretion plays an important role in group-cohesion, olfactory mate guarding [33,35], resource defence, and reproductive advertisement [34,36]. During the mating season, all mature males have large scrotal testes, and females exhibit a distinctly swollen, pink and everted vulva [37]. In contrast during autumnal reproductive quiescence, males have smaller testes that ascend into the body cavity, while females cease vulval swelling [37]. Sex steroid levels also follow distinct seasonal patterns in sexually mature badgers [31, 37, 38]. In males, testosterone levels are high in spring and summer, low in autumn, then peak during the winter mating season. In females, oestrone levels are high in spring, low in summer, peak in autumn then remain elevated for pregnant females in winter, but decline in non-pregnant females. As in all carnivores, male badgers have a baculum (os penis; [39]) that provides mechanical support during copulation, enabling prolonged intromission and mate-guarding through copulatory tying [40–41], which facilitates sperm transport [42] and helps induce ovulation [29].

Badgers produce one litter annually (mean litter size for study site = 1.4±0.06, range 1–4; 93% litters < 3 cubs [43]), born between mid-January—mid-March (76% born in mid-February in UK; [43]). Neonatal cubs are highly altricial and their eyes and ear canals do not open until 5 weeks [44]. Cubs are weaned at 6–8 weeks [45], coinciding with first emergence from their underground den, termed a sett [46], and become fully integrated into their social group at 14–16 weeks [46]. Growth rate depends on resource availability and competition for those resources, in terms of number of cubs born per social group and number of adults also present [47,48,49].

In high density areas (e.g. our site: 44.55 ± 5.37 (SE) badgers/km^2^; [43, 50]), cubs take longer to reach adult size [47, 48] and remain smaller than those living at lower density [47, 49]). Cubs start producing subcaudal gland secretion at approximately 4 months [33] but anoint themselves with secretion from adults (termed ‘scent-theft’) when much younger [46], signifying the importance of this secretion in badger sociality [35]. Reports of sexual maturity vary considerably, ranging from 9–12 months [51–52] to 18 months [33]; but most studies evade the exact age at puberty and simply state that female badgers cannot breed until 2 years old because of delayed embryonic implantation (reviewed in [44]). To-date no studies have investigated potential developmental heterochrony between individuals within the same population or cub cohort.

Here we describe for the first time male testosterone and female oestrone profile development for cubs, commencing from first capture at 3 months old and fully weaned, through to 28 months, when all individuals attain sexual maturity [51] and full adult size [48]. We also describe the ontological development of external genitalia morphology (EGM; degree of testicular descent and vulval swelling), baculum length, and testes volume. In other mammals somatic growth rates and sexual maturity vary among individuals within the same population [1,10], and so we additionally investigate if all cubs in our sample mature at the same rate, or follow different ontological strategies in terms of hormone profiles, skeletal growth and production of subcaudal gland secretion (as reported in other species: [16]) in relation to natal group size.

On account of delayed implantation we predict that puberty will occur around the second mating season (23–25 month) from birth; however as cub sex steroid levels develop they could also rise during the first mating season (11–13 months) following that individual’s birth. That is, we posit that not all individuals will attain puberty simultaneously. Furthermore, we predict that post-natal conditions may further modify inter-individual rates of juvenile development, thus affecting onset of puberty. We also posit that endocrinological maturation will be signified by the ontological development of reproductive organs and scent-glands.

## Materials and methods

### Badger trapping and sampling

Data were collected from a high-density (44.55±5.37 badgers/km^2^) badger population in Wytham Woods, Oxfordshire, UK (51°46:26 N, 1°19:19 W; for details see [43]), between 1995–2016, as part of an ongoing long-term research project. Badgers were trapped in every month except during the closed season (when trapping is prohibited during post-implantation, gestation, parturition and cub weaning) under the Protection of Badgers Act, 1992 (December-April); although, in some years, additional trapping was conducted under special license in December and early January before the end of the first pregnancy trimester [43]. The development of immature badgers could therefore be followed in: (1) *spring* (May/June) at end of the main mating period when cubs are fully weaned but adverse spring weather can impact cub growth [53]; (2) *summer* (July/August/September) during additional mating activity reported in other high-density badger populations [30] and the period of lowest food abundance [54, 55]; (3) *autumn* (October/November) during reproductive quiescence and highest food abundance; and (4) *winter* (December/January) during the main mating season when cold weather may affect thermal energy balance [56].

Following the methodology described in [57], traps were checked between 6.30–8.00 am and captured animals were transferred to holding cages and transported to a central field station before being sedated with 0.2 ml ketamine hydrochloride / kg body weight by intramuscular (quadriceps) injection [58–59]. In instances where animals were agitated, or would benefit from analgesia (i.e., those with naturally occurring bite wounds), we used ketamine / butorphanol combinations (20 mg/kg ketamine + 0.4 mg/kg butorphanol, i/m into the quadriceps). A typical procedure from initial sedation to completion of data collection and handling required 10–15 minutes per badger and processing of all captured badgers was typically completed before noon. After processing, badgers were given 3 hours (from the last procedure) to fully recover from sedation in a dark, quite recovery room, before being returned to their setts of capture. All badgers received a permanent unique tattoo at first capture (typically as cubs: [43, 55]), allowing individual (re-) identification (ID) and reliable aging.

### Somatic measurements and classification of external genitalia morphology

Head-body length (to the nearest 5mm), zygomatic arch width (to the nearest 1mm), and body weight (to the nearest 100g) were measured for all captured individuals, and a Body Condition Index (BCI) was calculated as log_10_(weight)/log_10_(body length). Subcaudal gland secretion was scooped out of the subcaudal pouch using a rounded stainless-steel spatula [33], and the volume estimated by eye to the nearest 0.05 ml. The spatula was disinfected between individuals using absolute ethanol [60]. External Genitalia Morphology (EGM) was categorised according to Sugianto et al. [37] in females as normal, intermediate or swollen vulva, and in males as ascended, intermediate or descended testes. Male baculum length, testes length, width and scrotal thickness were measured (in mm), and the testicular volume was calculated (in mm^3^) as (L x W x H) x 0.71, where L = testicle length—scrotal pinch, W = testicle width—scrotal pinch, and H = testicle width—scrotal pinch [61].

### Blood sampling and hormone measurements

Blood samples (n_males_ = 119; n_females_ = 63; chosen from archived data to represent each month—except the closed season, see above) were collected for endocrinological analyses via jugular venepuncture, using vaccutainer tubes (Becton-Dickinson) with K2-EDTA (ethylene diamine tetraacetic acid) anticoagulant. Sampling times were standardized to account for circadian variation in hormonal profiles [37–38], and blood samples were centrifuged within 30 minutes of sampling at 10°C for 10 min at 2,500 rpm/ 1470G. Plasma was transferred into Eppendorf tubes and frozen at -20°C immediately.

All sex steroid titres were analysed using Enzyme-immunoassays (EIA) at the Chester Zoo Endocrinology Laboratory, UK. Oestrone was measured in microtitreplates coated with polyclonal antiserum raised against oestrone EC R522 [62]. Plasma samples were un-extracted and used for measurement after dilution with assay buffer at the ratio of 1:10. Duplicate 20μl aliquots of oestrone standard (0.195–200 pg/well), diluted plasma, and quality controls were combined with 50μl oestrone glucuronide coupled to horseradish peroxidase (oestrone-glucuronide-HRP) as label, and incubated at room temperature for 2 hours. Plates were washed five times and blotted dry after incubation, followed by an addition of 100 μL peroxidase substrate solution (ABTS) to each well. Plates were covered and incubated at room temperature until the ‘0’ wells reached approximately 1.0 optical density and read at 405 nm using a Spectrophotometer Opsys MR (Dynex). Assay sensitivity at 90% binding was 3.1 pg. Intra-assay coefficients of variation (CV, calculated as the average value from the individual CVs for all of sample duplicates), were 8.21% (high) and 6.05% (low); inter-assay variation (repeated measurements of high and low-value quality controls across plates) was 13.96% (high) and 13.62% (low) respectively.

Testosterone was measured in microtitre plates coated with anti-testosterone R156/7 (OEM-Concepts, UK). Samples (un-extracted) were analysed by dilution in 1:4 assay buffer. Duplicate 50μl aliquots of testosterone standards (2.3–600 pg/well), samples and quality controls were then combined with 50μl horseradish peroxidase (testosterone-HRP) as label. After incubation in the dark at room temperature for 2 hours plates were washed 5 times and blotted dry, followed by addition of HRP-substrate (100 μL) to each well. Plates were covered and incubated at room temperature until the ‘0’ wells reached 1.0 optical density and were then read at 405 nm using a Spectrophotometer (Opsys MR; Dynex). Assay sensitivity at 90% binding was 1.6 pg. Testosterone intra-assay coefficients of variation were 14.69% (high) and 6.18% (low), and inter-assay variation of high and low-value quality controls was 9.15% (high) and 5.23% (low).

### Statistical analysis

All statistical analyses were performed using RStudio (0.99.896) and R (R-3.2.4). Patterns of residuals, normality, and mean variance for each model were checked using R diagnostic plots. Generalized Additive Models (GAM) were used to generate trend lines for sex steroid levels (males: testosterone, n = 119; females: oestrone, n = 63; as response) against age (3–28 months, as predictor) using a smoothing function (Tables A and B in S1 Dataset). A non-linear mixed model (random effect: badger identification/ tattoo number, ID) using the *nlme* and *sslogic* function was used to depict a growth curve (providing an asymptote value as output) for baculum length (as response) against age (3–28 months, n = 773, as predictor; Table C in S1 Dataset). To determine the age at which the baculum ceased to grow, the percentage of predicted baculum length towards the asymptote (in the adult population) was calculated. Trend lines for testes volume (n = 597; Table C in S1 Dataset) and subcaudal secretion volume (n_male_ = 1233, Table C in S1 Dataset; n_female_ = 1284, Table D in S1 Dataset) as responses were generated against age (3–28 months, as predictor) by fitting a GAM model. Interactions between proportions of EGM (males: descended, intermediate, ascended testes, n = 1136, Table E in S1 Dataset; females: normal, intermediate, swollen vulva, n = 1174, Table F in S1 Dataset, as response) with age (3–28 months, as predictor) were analysed using a Chi-square test.

### Developmental heterochrony

#### Endocrinology and EGM

GAM average trends (above) provided a legitimate basis to ascertain hormonal heterochrony in both sexes, where some cubs reached puberty earlier than others. Individuals were categorized by whether they fell above or below (high vs low category) the GAM line benchmark at a certain age, providing discrete two endocrinological groups, or phenotypes. Individuals within the convidence interval (grey area) were also grouped based on the average GAM line. However, endocrinological sample sizes were limited, and so to enhance analytical power we also repeated all analyses conducted on hormone-based groups (below) to EGM-based groups at the age of 11 months. That is, in addition to comparing cubs with high vs low sex-steroid levels we also compared male cubs with ascended vs descended testes and female cubs with a normal vs swollen vulva; excluding intermediate conditions in both sexes to avoid ambiguity.

#### Somatic growth

To determine potential concurrent differences in physical development at the point of hormonal divergence we subsequently compared head-body length, zygomatic arch width, BCI, and subcaudal secretion volume between these two endocrinological groups using a linear model (including year as a factor to account for established inter-annual variation in growth patterns: [63, 48]; Tables G and H in S1 Dataset). If significant differences were found in any of these morphometric measures, differences in head-body length and zygomatic arch width between adult size (above 28 months) compared to the size at the age at which divergence occurred were calculated for all individuals, and then compared between the endocrinological groups to determine heterochronous residual growth (Table I in S1 Dataset). We then constructed growth curves for individuals captured repeatedly in each group based on the rates of increase in head-body length (which provide a reliable indicator for overall skeletal growth and development of badger cubs [48]), employing a non-linear mixed model (random effect: ID) using the *nlme* and *sslogic* function (Table J in S1 Dataset). When individuals were not recaptured at this precise target-age (within the 28 months period), we used the closest recapture point available for these analyses. All these analyses described above were also conducted on the EGM-based groups (differences in physical development: Tables K, L, M in S1 Dataset; differences in residual growth: Table N in S1 Dataset; differences in growth curves: Table O in S1 Dataset).

#### Social factors affecting the timing of puberty

In addition, we investigated if social factors/ intra-specific competition affected the onset of sexual maturity by comparing hormone levels at the age when these phenotypic groups diverged against the total number of adults and cubs in that cub’s natal group and natal sett (as some groups utilize several setts: [64]), with year included as a factor (hormone-based groups: Tables G and H in S1 Dataset; EGM-based groups: Tables K and L in S1 Dataset). Annual badger group residency was determined according to the rules in Annavi et al. [50]; see also Sugianto et al. [48]).

## Results

### Endocrinological changes during the first 28 months

**Fig 1:**
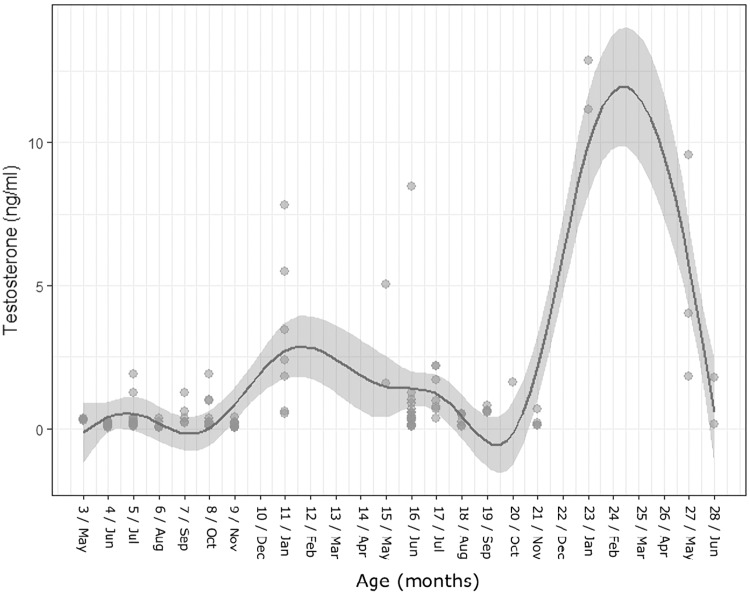
Testosterone levels (ng/ml) in males aged 3–28 months. GAM model average trend line for testosterone levels against age (solid line) and the confidence interval (grey area) using a smoothing function.

As predicted, throughout their first summer, all cubs had significantly lower sex-steroid levels than did adults. However, at the age of 11–12 months, i.e. coincident with the first population mating season cubs lived through, male cubs showed a small peak in testosterone (GAM: Edf = 8.631, R-sq.(adj) = 0.566, GCV = 2.230, Deviance explained = 59.9%, p<0.001, Fig 1). Male cubs then followed the seasonal pattern of testosterone levels typifying adults (high in spring and summer, and low in autumn: [38]), that reached adult levels with a pronounced peak during their second mating season (22–28 months).

**Fig 2:**
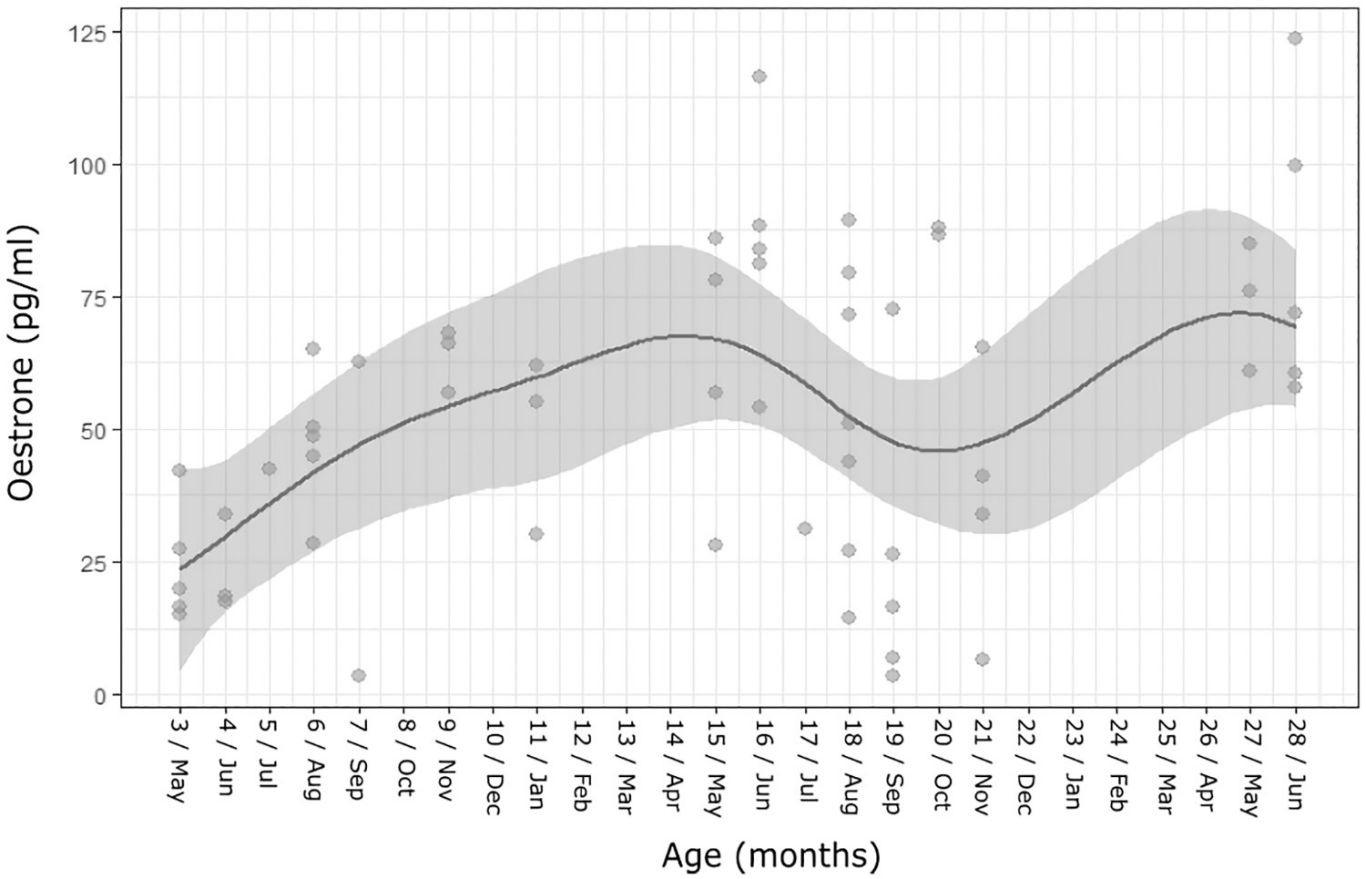
Oestrone levels (pg/ml) in females aged 3–28 months. GAM model average trend line for oestrone levels against age (solid line) and the confidence interval (grey area) using a smoothing function.

In females, oestrone levels increased gradually from 3 to 11 months, at which point they almost reached adult levels (GAM: Edf = 5.218, R-sq.(adj) = 0.216, GCV = 733.1, Deviance explained = 28.2%, p = 0.009, Fig 2). Patterns then followed the seasonal oestrone pattern typical for adults, with relatively high levels in spring (13–16 months) then decreasing in summer (17–19 months), remaining low during autumn (20–21 months, reproductive quiescence) and winter (22 months, December: implantation) after which point they increased again towards spring (27–28 months) to reach adult levels (73.28±28.06 pg/ml; [31]). Nevertheless, inter-individual variation among females was considerable during the second summer (months 15–20), i.e., from the end of the first mating season they lived through until autumnal reproductive quiescence.

### Changes in external genitalia during the first 28 months

**Fig 3:**
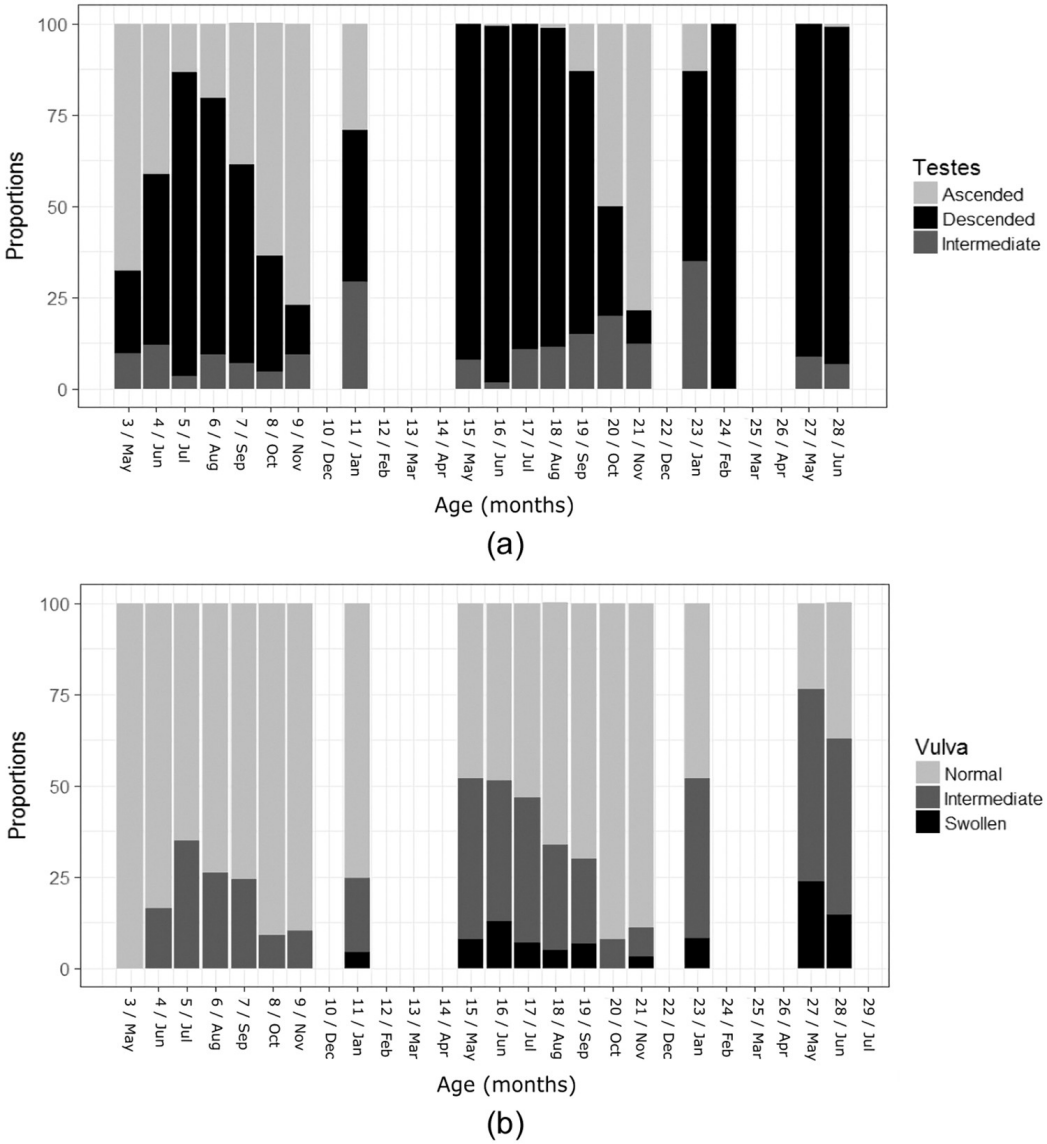
EGM changes in males (a; ascended, intermediate, descended) and females (b; normal, intermediate, swollen) aged 3–28 months.

In both sexes, there was a significant interaction between EGM and age (in months) (males_n = 1136_: X^2^ = 937.04, df = 36, p<0.001; females_n = 1174_: X^2^ = 418.47, df = 34, p<0.001; Fig 3a and 3b). The majority of male cubs had scrotal (i.e., fully descended) testes for the first time at 5–6 months (83.3% and 70.4% respectively), while during their first autumn (8–9 months) the majority had ascended testes (63.6% and 77.1% respectively). During the first mating season they lived through (January at 11 months), the largest proportion (41.5%) of male cubs had descended testes, while both ascended and intermediate proportions were each 29.3%. During the following spring (15 months) and summer (19 months) the majority of males (94.8% and 82.9%, respectively), had descended testes and followed the adult seasonal pattern thereafter.

In females, the earliest vulval swellings (4.4% swollen; 20% intermediate vulva) were recorded at 11 months during the first mating season they lived through (Jan). The proportions of females with intermediate and swollen vulva increased during the next spring-summer (15–19 months; intermediate: spring = 41.4%, summer = 30.5%; swollen: spring = 10.3%, summer = 6.2%) then decreased in autumn (20–21 months; intermediate: 7.8%, swollen: 1.5%). During their third spring (27–28 months), the highest percentage of female cubs had either intermediate (50.4%) or fully developed vulval swelling (19.2%), congruent with adult states [37].

**Fig 4:**
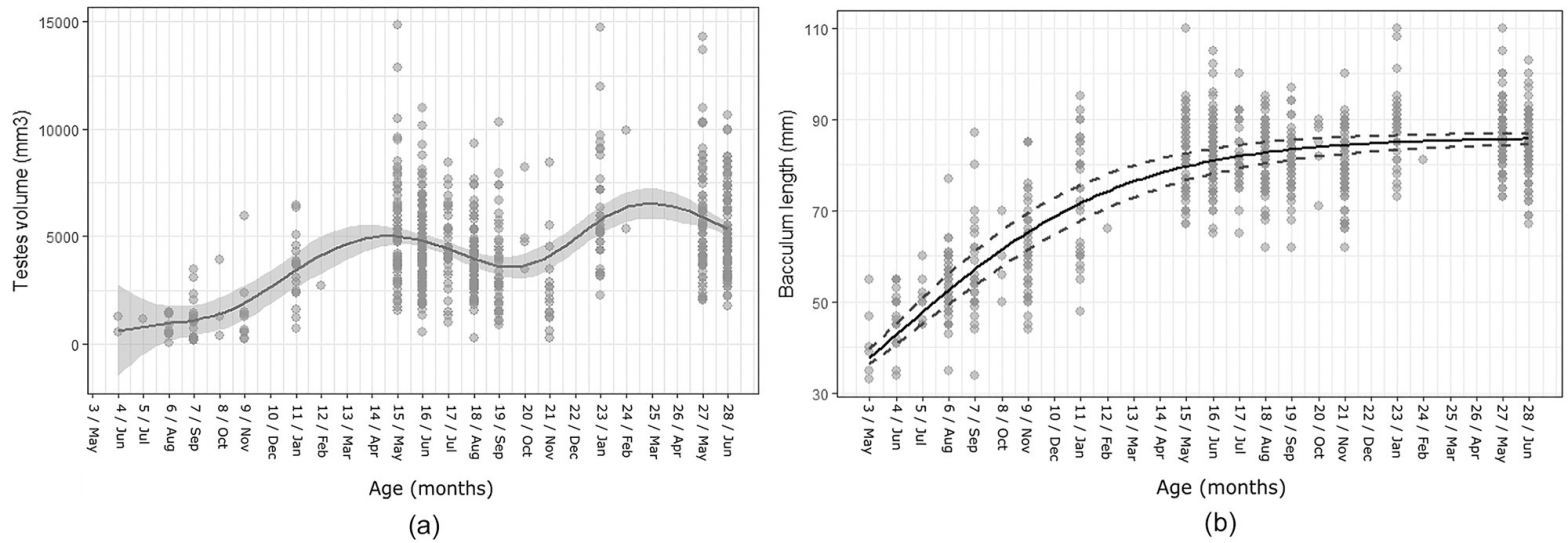
(a) Testes volume (mm^3^) in males aged 3–28 months. GAM model average trend line for testes volume against age (solid line) and the confidence interval (grey area) using a smoothing function. (b) Baculum length growth curve at age of 3–28 months in males. Growth curve average trend against age (solid line) and confidence interval (dotted line).

In males, testicular volume started to increase markedly at the age of 11 months (from 848.69±475.20 mm^3^ at the age of 4–6 months_n = 12_; 1331.27±1289.97 mm^3^ at the age of 7–9 month _n = 32_; to an average of 3449.73±1572.04 mm^3^ at 11 months _n = 18_) and peaked during the first mating season they lived through (5326.45±2674.88 mm^3^, n = 60; winter-early spring; GAM: Edf = 6.921, R-sq.(adj) = 0.242, Deviance explained = 25.1%, p>0.001, Fig 4a). Average testicular volume then decreased towards an autumnal minimum at 20–21 months (3209.42±2283.41 mm^3^, n = 20) and followed the adult seasonal pattern thereafter, reaching a slightly higher peak (5698.26±2409.85 mm^3^, n = 187) in the second mating season (22–28 months), with sizes comparable to adult values thereafter (winter: 6650.82 mm^3^, spring: 5776.31 mm^3^: [37]). Male baculum length increased consistently month by month for the first year, after which baculum growth rates slowed and reached 99% towards the asymptote of 86.03 mm predicted by our model at 23–24 months (Fig 4b).

### Subcaudal gland activity during the first 28 months

**Fig 5:**
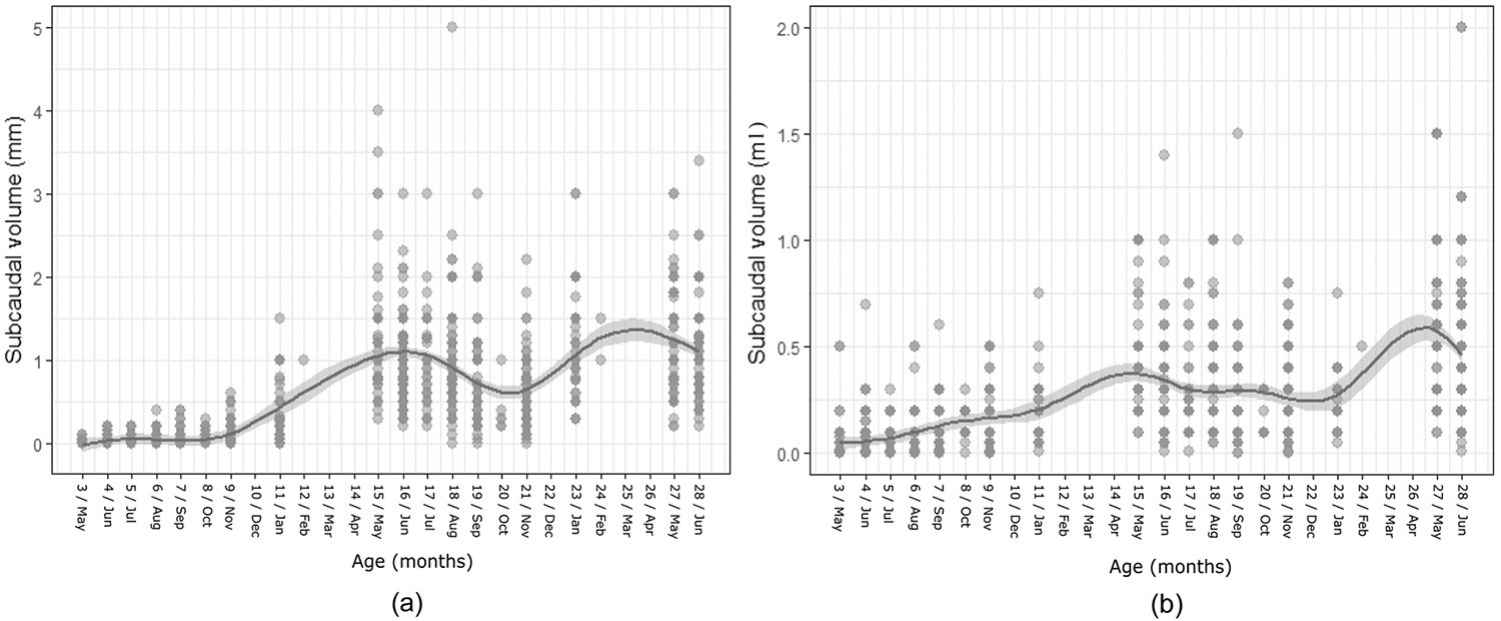
Subcaudal secretion volume (ml) changes in males (a) and females (b) aged 3–28 months. GAM model average trend line for subcaudal secretion volume against age (solid line) and the confidence interval (grey area) using a smoothing function.

Both sexes started producing subcaudal gland secretion at a similar age (detected at first capture at 3 months; Fig 5a and 5b). Nevertheless, secretion volume was very low (unmeasurable traces in males and 0.04±0.09 ml in females _n = 72_) and increased only slowly towards their first mating season at age 11 months (0.38±0.36 ml in males_n = 41_ and 0.19±0.13 ml in females_n = 47_). Thereafter, secretion volume increased substantially in both sexes (GAM: male: Edf = 8.45, R-sq.(adj) = 0.56, GCV = 0.18, Deviance explained = 56.2%, p<0.001, Fig 5a; female: Edf = 8.83, R-sq.(adj) = 0.37, GCV = 0.04, Deviance explained = 38.2%, p<0.001, Fig 5b). In male yearlings, secretion volume peaked in spring-summer (13–18 months, 1.04±0.61 ml, n = 292), decreased towards an autumn-minimum (20–21 months; 0.56±0.39 ml, n = 97) and peaked again (with higher secretion volume) in their second winter-spring (23–28 months; 1.17±0.58 ml, n = 210), following the typical seasonal pattern and secretion volume of adults (average values for spring = 1.06±0.67 ml, n = 1004; summer = 0.92±0.66 ml, n = 1059; autumn = 0.60±0.50 ml, n = 790; winter = 0.90±0.60 ml, n = 347). Female yearlings showed a first small peak in secretion their second spring (13–16 months; 0.35±0.24 ml, n = 150), after which volume decreased slightly during summer-autumn (17–19 months, 0.32±0.35 ml, n = 189 and 20–21 months, 0.27±0.17 ml, n = 110), but then started to increase again at the end of winter (24 months), peaking at an average of 0.52±0.41 ml in spring (27–28 months) and following the adult pattern thereafter (average values for spring = 0.41±0.34 ml, n = 1287; summer = 0.33±0.32 ml, n = 1286; autumn = 0.25±0.24 ml, n = 933 ml; winter = 0.24±0.26 ml, n = 207).

### Evidence for two cub phenotypes: Early and late developers

#### Endocrinological evidence for early and late developers

In both sexes, some individuals appeared to reach puberty earlier than others evidencing the existence of two endocrinological phenotypes: early and late developers (Figs 1 and 2). These were evident from sex-steroid levels clustering into two distinct trait types according to the GAM line benchmark (for detailed results see Tables 1 and 2). However, the age at which these early and late development phenotypes became apparent differed between male and female cubs. Some males (3/7 = 42.9%) cubs reached puberty during their first year (HT, n = 3, testosterone levels above the GAM line at 11 months of age), while the remainder reached puberty during their second year (LT, n = 4, testosterone levels below the GAM line at 11 months, reaching pubescent levels at 22–28 months of age; Fig 1). In contrast, in females, these endocrinological phenotypes diverged at age 15–18 months (younger cubs either had more unified levels or sample sizes were too small to signify a difference; Fig 2), where some females had above-average oestrone levels (HO, n = 9, oestrone above the GAM line) and some below-average levels (LO, n = 8, oestrone below GAM line). In both sexes, these endocrinological phenotypes manifested independent of calendar year.

**Table 1:** Differences in somatic development between early and late developers from endocrinological and EGM grouping in male cubs.

Grouping based on hormone levels in male cubs					
Endocrinological and somatic difference (at 11 months) between groups					
Endocrinological and somatic parameter	Groups based on hormone level				Linear model statistics (accounting for year as covariate)
	HT		LT		
	Avg + std	N	Avg + std	N	
Testosterone level (ng/ml)	5.58±2.19	3	1.15±1.14	4	F_1,4_ = 9.534, p = 0.037
Zygomatic arch (mm)	83.67±2.52	3	76.00±4.32	4	F_1,4_ = 6.333, p = 0.066
Head body length (mm)	656.67±23.09	3	610±23.45	4	F_1,4_ = 8.448, p = 0.044
Body Condition Index	0.31±0.01	3	0.29±0.02	4	F_1,4_ = 5.831, p = 0.073
Subcaudal secretion volume (ml)	0.33±0.32	3	0.14±0.08	4	F_1,4_ = 1.164, p = 0.341

Residual growth (11–28 months) difference between groups					
Somatic parameter	Groups based on hormone level				Linear model statistics (accounting for year as covariate)
	HT		LT		
	Avg + std	N	Avg + std	N	
Zygomatic arch (mm)	5.67±0.58	3	12.25±0.96	4	F_1,4_ = 180.437, p<0.001
Head body length (mm)	28±26.96	3	77.5±19.36	4	F_1,4_ = 102.892, p<0.001

Grouping based on EGM in male cubs					
Somatic difference (at 11 months) between groups					
Somatic parameter	Groups based on EGM				Linear model statistics (accounting for year as covariate)
	DT		AT		
	Avg + std	N	Avg + std	N	
Zygomatic arch (mm)	83.11±3.82	9	79.40±4.33	10	F_1,16_ = 1.969, p = 0.180
Head body length (mm)	668.57±24.21	14	632.08±51.28	12	F_1,22_ = 9.389, p = 0.006
Body Condition Index	0.32±0.01	13	0.30±0.03	12	F_1,22_ = 10.611, p = 0.004
Subcaudal secretion volume (ml)	0.54±0.44	17	0.13±0.11	11	F_1,25_ = 10.730, p = 0.003

Residual growth (11–28 months) difference between groups					
Somatic parameter	Groups based on EGM				Linear model statistics (accounting for year as covariate)
	DT		AT		
	Avg + std	N	Avg + std	N	
Zygomatic arch (mm)	6.50±3.25	8	11.60±4.12	10	(F_1,15_ = 7.805, p = 0.014
Head body length (mm)	33.21±15.14	14	70±38.79	12	F_1,23_ = 12.620, p = 0.002

**Table 2:** Differences in somatic development between assumed phenotypes from endocrinological and EGM grouping in female cubs.

Grouping based on hormone levels in female cubs					
Endocrinological and somatic difference (at 15–18 months) between groups					
Endocrinological and somatic parameter	Groups based on hormone level				Linear model statistics (accounting for year as covariate)
	HO		LO		
	Avg + std	N	Avg + std	N	
Oestrone level (ng/ml)	86.06±12.72	9	38.31±15.32	8	F_1,14_ = 48.283, p<0.001
Zygomatic arch (mm)	80.44±3.81	9	81.29±3.15	7	F_1.13_ = 0.219, p = 0.647
Head body length (mm)	672.22±23.6	9	662.5±35.15	8	F_1,14_ = 0.700, p = 0.417
Body Condition Index	0.3±0.02	9	0.3±0.04	8	F_1,14_ = 0.014, p = 0.909
Subcaudal secretion volume (ml)	0.48±0.42	8	0.9±1.46	5	F_1,10_ = 0.273, p = 0.613

Grouping based on EGM in female cubs					
Somatic difference (at 15–18 months) between groups					
Somatic parameter	Groups based on EGM				Linear model statistics (accounting for year as covariate)
	SV		NV		
	Avg + std	N	Avg + std	N	
Zygomatic arch (mm)	83.41±2.22	22	82.85±3.96	124	F_1,143_ = 0.446, p = 0.505
Head body length (mm)	678.1±18.54	21	675.53±26.59	148	F_1,166_ = 0.188, p = 0.665
Body Condition Index	0.3±0.01	21	0.3±0.02	147	F_1,165_ = 0.051, p = 0.822
Subcaudal secretion volume (ml)	0.36±0.22	21	0.32±0.21?	142	F_1,160_ = 0.574, p = 0.450

Somatic difference (at 11 months) between groups					
Somatic parameter	Groups based on EGM				Linear model statistics (accounting for year as covariate)
	SV		NV		
	Avg + std	N	Avg + std	N	
Zygomatic arch (mm)	83.5±7.78	2	78.2±3.79	25	F_1,24_ = 3.105, p = 0.091
Head body length (mm)	675±21.21	2	645.88±37.43	34	F_1,33_ = 1.247, p = 0.272
Body Condition Index	0.32±0.02	2	0.31±0.03	34	F_1,33_ = 0.764, p = 0.388
Subcaudal secretion volume (ml)	0.2±0.0	2	0.18±0.14	33	F_1,33_ = 0.221, p = 0.641

#### Differences in somatic development between early and late developer phenotypes

**Males**. Comparing the extent of somatic development between the two endocrinological phenotypes (at age 11 months), HT males (n = 3) had significantly larger head-body length than LT cubs (n = 4), were larger overall, and showed a near significant difference in zygomatic arch width and BCI; but did not differ in the volume of subcaudal gland secretion they produced.

To test whether the early endocrinological development of individuals assigned to the HT group was a product of more rapid development to adult size (thus closer to being fully developed, sexually mature adults) and were not just simply larger cubs, we compared differences in head-body length and zygomatic arch width above 28 months (i.e., fully developed adults) with those of cubs assigned to HT- and LT endocrinological groups at 11 months. Difference in these measurements between HT-cubs and adults was significantly smaller than between LT-cubs and adults, confirming that HT-cubs were closer to being fully developed adults (see Table 1).

**Fig 6:**
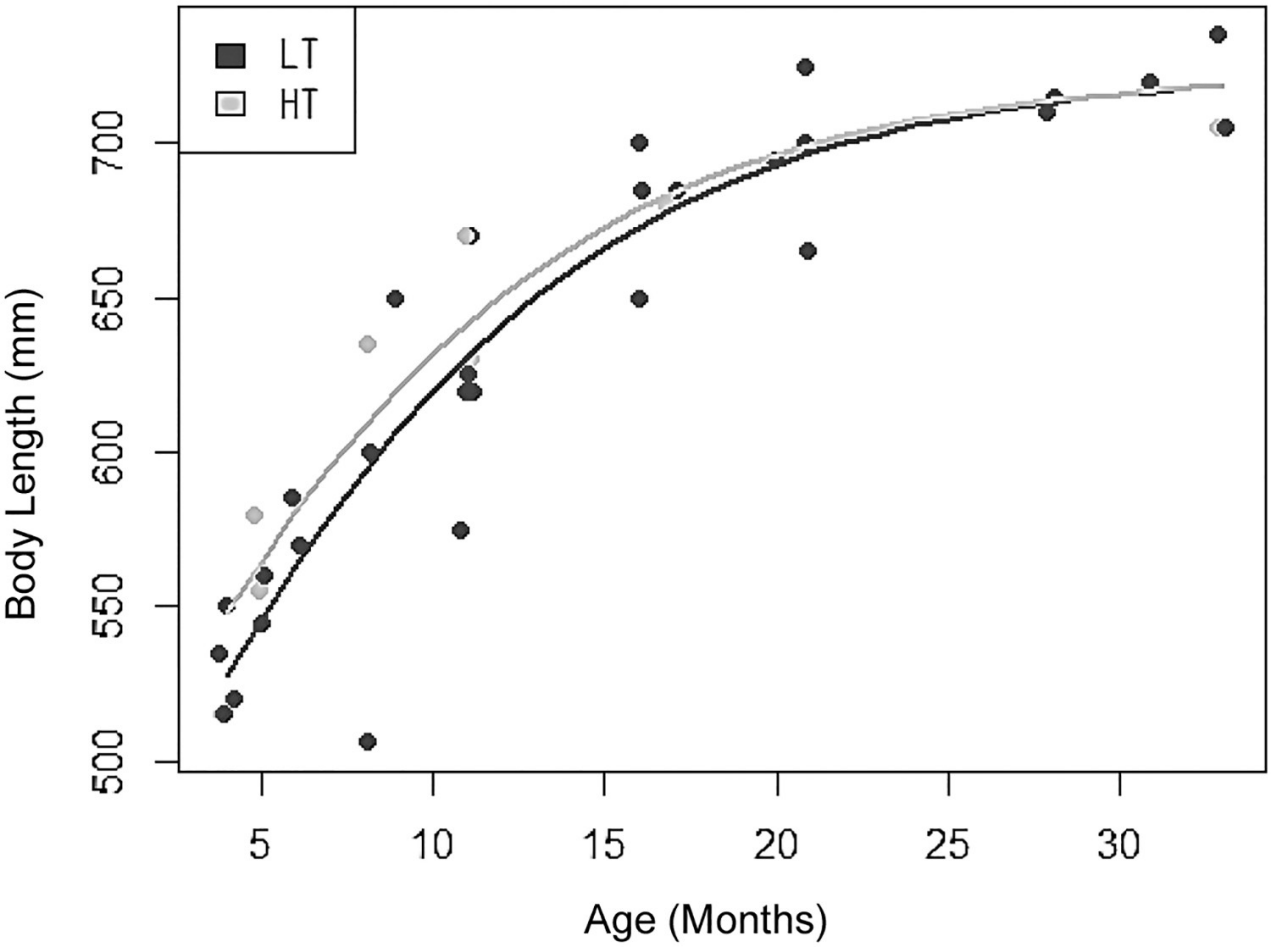
Body length growth curve of HT (open circles) and LT (solid black circles) groups, age: 4–33 months.

For males, comparing the growth curves of HT (8 repeat-measures over 33 months from 3 individuals) and LT types (32 repeat-measures over 33 months from 4 individuals) revealed a trend (albeit non-significant, likely due to limited sample sizes) for LT-cubs to grow more slowly than HT-cubs, despite ultimately reaching similar adult head-body lengths (X^2^ = 1.873, df = 7, p = 0.392; non-linear mixed model), where HT had already reached 95% of the maximum head body length at the age of 11 months, while LT reached this percentage later at the age of 14 months. This difference in body size unified at the age of 19–20 months, when growth rates of HT- and LT-males equalised (Fig 6).

We repeated these analyses based on the degree of testicular descent at 11 months, comparing cubs that had fully descended testes (DT, n = 18, assumed to have reached puberty) with those that had ascended testes (AT, n = 13, assumed to have not reached puberty). Overall, these showed differences in somatic development consistent with the two endocrinological phenotypes (for detailed results see Table 1). DT cubs were considerably longer (head-body length), were in significantly better body condition/BCI, and had significantly more subcaudal secretion at 11 months than AT males; but no difference was found in zygomatic arch width. DT cubs also had significantly smaller adult vs cub differences in head-body length and zygomatic arch width than AT cubs, which corroborated that (like HT-males) they were closer to adulthood.

**Fig 7:**
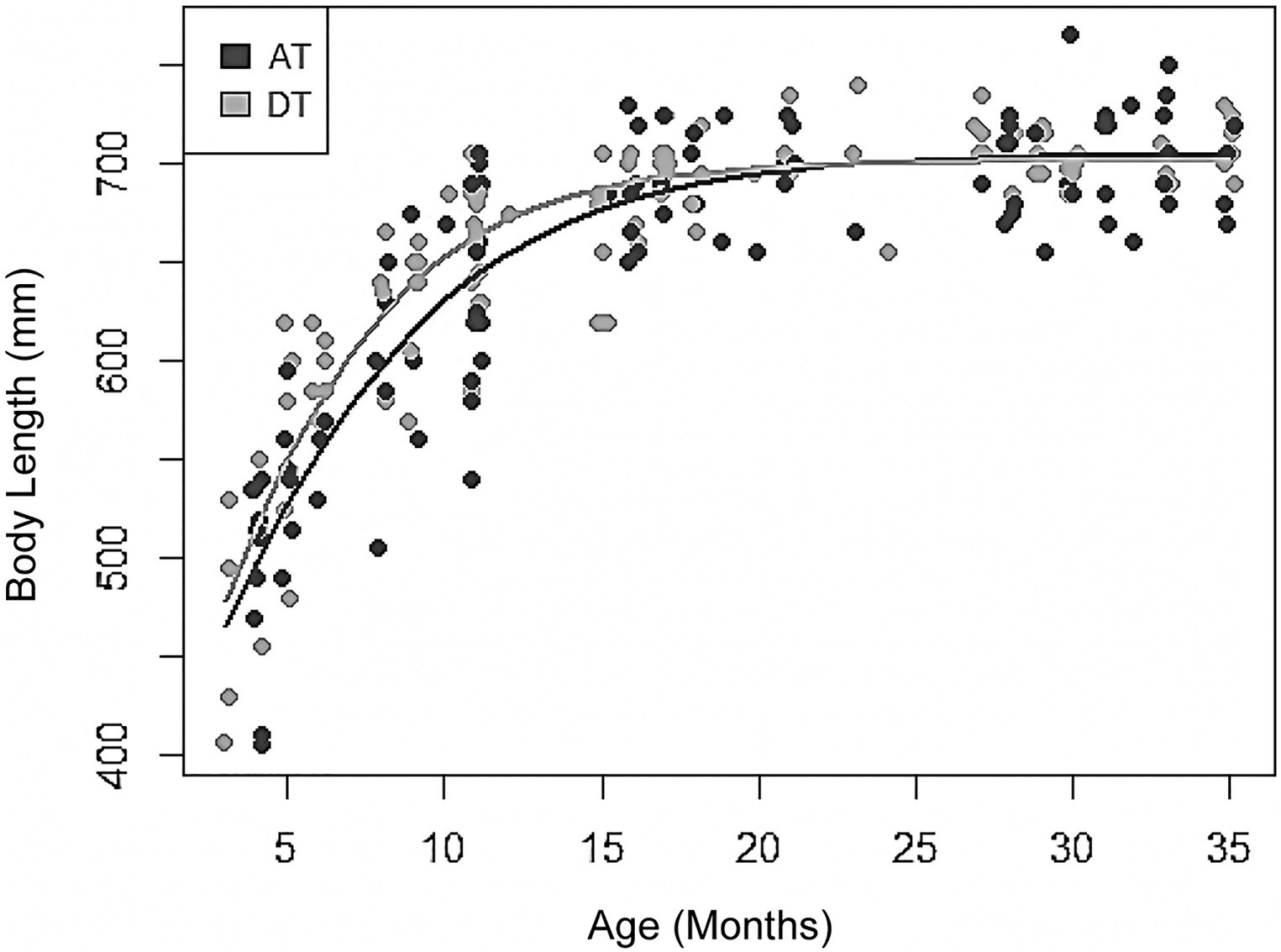
Body length growth curve of descended testes (DT) cubs and ascended testes (AT) cubs, age: 3–35 months.

Mirroring the trend found in the endocrinological phenotypes there was a significant difference in the growth curves of these two EGM-based groups (Fig 7; X^2^ = 10.087, df = 8, p = 0.018), with DT cubs (117 repeat-measures taken over 35 months from 18 cubs) growing faster, and reaching adult size earlier than AT cubs (99 repeat-measures taken over 35 months from 13 cubs). At 19–20 months this difference unified and growth rates of DT and AT cubs equalised.

**Females**. In contrast, for females, we detected no (significant) differences between any of the somatic parameters, nor for subcaudal gland volume, when comparing between the early (n_HO_ = 9) and late (n_LO_ = 8) developing endocrinological phenotypes (see Table 2). To ensure that the somatic similarity found between the two endocrinological phenotypes was not an artefact of the smaller sample size for our endocrinological dataset, we used vulva category at the age 15–18 months as the criteria defining stage of development. Again we found no significant differences (see Table 2) in any somatic parameters nor subcaudal gland volume between SV cubs (n = 24) and NV (n = 152), evidencing that both vulva condition types exhibited similar body size by age.

Because some female cubs first exhibit vulval swelling at 11 months (n_SV_ = 2, n_NV_ = 34), we repeated these analyses (see Table 2) at this younger age, but again found no significant difference in BCI, head-body length, nor subcaudal gland volume, although we did detect a slight difference in zygomatic arch width between these groups.

#### Social factors influencing the timing of puberty

At 11 months (see Table 3) testosterone levels in male cubs tended to be lower for individual born into larger natal social groups and setts, albeit without statistical significance, although the high R-value (0.53 and 0.63 for resident adults in natal social group and sett respectively) evidences a strong correlation, where non-significance is likely due to low sample sizes. A similar interaction was suggested by an even higher R-value with number of other cubs present in the natal social group and sett (0.76 and 0.85 for other resident cubs in natal social group and sett repectively). That is, cubs born/ growing up in smaller social groups and/ or setts seem more likely to be early developers than those born/ growing up in larger groups/ setts. Using degree of testicular descent at 11 months instead of the endocrinological phenotypes, however, suggested no trend (see Table 3).

**Table 3:** Social factors affecting the timing of puberty in male and female cubs

Social factors affecting timing of puberty in male cubs (11 months)		
Social factor	Testosterone	EGM
Number of adults in natal social group	R = -0.53; F_1,4_ = 1.407, p = 0.301, n = 7	R = -0.39; F_1,26_ = 1.052, p = 0.314, n = 17
Number of adults in natal sett	R = -0.631; F_1,3_ = 1.446, p = 0.316, n = 6	R = 0.31; F_1,23_ = 1.994, p = 0.171, n = 17
Number of cubs in natal social group	R = -0.763; F_1,3_ = 3.527, p = 0.157, n = 6	R = 0.36; F_1,22_ = 2.543, p = 0.125, n = 17
Number of cubs in natal sett	R = -0.851; F_1,4_ = 5.359, p = 0.082, n = 7	R = 0.46; F_1,17_ = 2.555, p = 0.128, n = 16

Social factors affecting timing of puberty in female cubs (15–18 months)		
Social factor	Oestrone	EGM
Number of adults in natal social group	R = -0.157; F_1,14_ = 0.039, p = 0.846, n = 29	R = -0.1; F_1,172_ = 0.272, p = 0.603, n = 175
Number of adults in natal sett	R = -0.116; F_1,14_ = 0.016, p = 0.901, n = 26	R = -0.23; F_1,165_ = 2.281, p = 0.133, n = 168
Number of cubs in natal social group	R = -0.267; F_1,14_ = 0.430, p = 0.523, n = 25	R = 0.02; F_1,144_ = 0.002, p = 0.963, n = 147
Number of cubs in natal sett	R = -0.092; F_1,13_ = 0.052, p = 0.823, n = 20	R = -0.02; F_1,102_ = 0.005, p = 0.944, n = 105

Female cubs showed only very weak negative relationships between oestrone levels at the age of 15–18 months with the number of adults resident in their natal social group and sett, as well as the number of other cubs in their natal social group and sett. When cubs were categorised on the basis of their EGM at the age of 15–18 months, again no effects were found (see Table 3).

## Discussion

We demonstrate that, in badgers, puberty begins in both sexes at ca. 11 months of age, when cubs develop similar seasonal sex-steroid patterns to adults. Furthermore, in both sexes, all parameters that support reproductive activity and mating-associated behaviours, such as external genitalia morphology (and in males also testes volume), and subcaudal gland secretion volume, show similar developmental patterns to sex steroid levels, further corroborating the onset of puberty [1]. The increase of sex-steroid levels likely triggers changes in EGM [37], as well as in the activity of species-specific subcaudal glands important in the context of reproduction and sexual advertisement [65]. Nevertheless, cub hormone titres typically remained lower than those reported for adults (males: [38]; females: [31]) until their second mating season (22–26 months).

In male cubs, testosterone levels remained low and exhibited no seasonal variation until the first winter mating season, when they started to increase, reaching a smaller peak than in adults [38]. Levels then remained elevated until the end of the mating season, and followed the seasonal pattern typifying adults thereafter. By the time male cubs reached the second population breeding season, their testosterone titres had reached higher levels compared to the first mating season, in accord with adults [38]. Nevertheless, this larger peak during the second mating season was driven by fewer datapoints than the first smaller peak, due to far fewer captures during the closed season (datapoints in January were only obtained through limited additional trapping under special governmental license). Baculum growth also reached the population-based asymptote by the cubs’ second mating season, indicating the completion of sexual development. These findings support the hypothesis by Whelton and Power [39], who measured baculum length post-mortem in road kill and culled badgers, positing that the observed abrupt decrease in baculum growth rate coincides with sexual maturity; although their post-mortem study was unable to verify this conclusion through endocrinological measurements.

In contrast, in females, we observed a gradual increase in oestrone from 3–11 months (May-January) without any noticeable seasonal variation. After 11 months, however, cub oestrone levels started to follow the same seasonal pattern as adults [31], with high levels in spring and low levels in summer. Nevertheless, although adult females oestrone levels typically increase in autumn and remain elevated until blastocyst implantation in December [31], cub oestrone levels remained low until the next mating season (January), implying that—counter to observations from other, low-density studies [51]—no female cubs in our dataset were capable of mating successfully during their first year, corroborating genetic results from our study population reported previously [66].

Inter-individual variation in plasma sex-steroid levels was, however, considerable among same-age cubs of either sex, and we observed two distinct groups: early and late developers. We infer these to qualify as distinct phenotyptic response types, given the potential fitness advantage of early maturity [67], but set against the reality of resource limitation and social stress in wild populations precluding all individuals from engaging in the maximal developmental response [47]. In male cubs, endocrinological profiles and EGM indicated that some had reached sexual maturity at 11 months, while the remaining cubs likely achieved this only during their second winter. At this time all male cubs showed similarly high testosterone peaks comparable to adult levels, and thus puberty had concluded. Similarly, there was substantial variation in the proportion of the final body length males had achieved by 11 months, mirroring the differences in testosterone levels observed during this period. This increase in head body length ceased by age ca. 18 months (99% towards asymptote; [48]) which is also the age at which body lengths equalised when dividing males according to testes descent (DT and AT as well as endocrinological groups HT and LT). Nevertheless, when we cross-referenced against assigned paternity data, none of the individuals exhibiting high hormone levels at 11 months (HT = 3 individuals) were assigned cubs in the following spring. For female cubs, variation in oestrogen levels was high during their second summer (May-Sept/ Oct), indicating that not all females reached sexual maturity at the same age, but that puberty onset varied between 15–18 months.

The existence of different ontological phenotypes (i.e., early and late developers) has been described in other members of the Mustelidae, and has been linked to body size and species-specific life history traits [25] For example, in captive sables (*Martes zibellina*) a small proportion of all individuals are reported to reproduce at 15 months of age, whereas the majority (80%) of males and females starts breeding at the age of 27 months [25]. Our results support observations from badgers in Sweden, where spermatozoa were first recorded in male cubs at 12 months [51]. Nevertheless, in this Swedish low-density population the majority of males reached puberty in their first year, and only a minority of males did not produce spermatozoa until their second summer, or even winter (24 months: [51]). In our high-density population, in contrast, most males reached puberty in their second year.

Overall, our results support the hypothesis that mammals typically need to reach a threshold body size for sexual maturation [10]. Thus, the age at which puberty occurs is likely not only influenced by the gene load of the individual but also by ecological factors such as access to food (affected by weather/ climate), competition, and differences in demographic factors [1]. Resource availability tends to vary across time and space [68], and access to resources is further constrained by the number of competing conspecifics present leading to social stress [64]. Consequently, energy budgets can differ substantially between individuals even within a single population/ year, with the potential to drive considerable variability in the timing of sexual maturity [1]: Individuals that develop under poor nutritional conditions, or subject to more social stress resulting from competition, usually reach sexual maturity at slower rates [11–13]; as implied by our observation that male cubs born into more populated setts and social groups tended to be biased toward the late developer phenotype.

Generally, in mammals (especially those with polygynous mating systems), males tend to grow more quickly than females [69], and ultimately attain a larger body-size (i.e., dimorphism; see Badyaev [70]). Our data show that this is also the case in badgers (see also Sugianto et al. [48]; NB, our measurements were made after weaning, thus obviating differential maternal investment effects; [71]). Consequently, males are likely also more vulnerable to resource limitation and social competition, with the potential to impact their degree of development by the end of their first year, explaining the observed delay in puberty in more populated social groups [72–73]. Our findings are congruent with those for female brown bears (*Ursus arctos*), where adult body size shows a negative relationship with population density [74]: female bears are larger and grow faster in areas with better environmental conditions, but when subject to higher resource competition females are smaller and grow more slowly. As in our badger study, bears have been shown to compensate for slower growth rates by delaying reproductive activity [74] at the potential cost of lower lifetime reproductive success [75]. Similar negative correlations between population density and individual growth rate has been reported in the northern fur seal (*Callorhinus ursinus*; [76]), polar bears (*Ursus maritimus*; in terms of smaller juvenile body length [77], and adult body size [78]), and in American black bears (*Ursus americanus*; with lower yearling weight; [79]). Similarly, in farmed red deer (*Cervus elaphus*), Blanc and Thériez [80] found that under high stocking density the growth rate of subordinate females was 2.5 times slower, and average daily weight gain of all juvenile hinds was significantly impaired. Demographic effects have also proven to affect the onset of maturation in female baboons (*Papio cynocephalus*), where first mentruation was earlier in smaller groups where individuals experience less social stress and competition [81].

We thus conclude that the asynchronous timing of puberty, leading to two heterochronous phenotypes, can occur even within a single population, and is likely caused by individuals attaining the required minimum body size according to different rates. Ultimately, capacity to breed at a young(er) age can have profound effects on life-history trade-offs (see [82] with early-life success often being critical to an individual’s fitness [67], which can substantially enhance population growth rate [83].

## Supporting information

S1 Dataset Testosterone and oestrone levels, subcaudal gland secretion volume, morphological measures, hormone-based groups, EGM-based groups, adult-cub size difference, and cub growth curves. (XLSX)
